# Fabrication and Characterization of Monolithic Integrated Three-Axis Acceleration/Pressure/Magnetic Field Sensors

**DOI:** 10.3390/mi15030412

**Published:** 2024-03-19

**Authors:** Ying Wang, Yu Xiao, Xiaofeng Zhao, Dianzhong Wen

**Affiliations:** 1 The Key Laboratory of Electronics Engineering, College of Heilongjiang Province, Heilongjiang University, Harbin 150080, China; 1213047@s.hlju.edu.cn (Y.W.); 2221700@s.hlju.edu.cn (Y.X.); wendianzhong@hlju.edu.cn (D.W.); 2 Heilongjiang Provincial Key Laboratory of Micro-Nano Sensitive Devices and Systems, Heilongjiang University, Harbin 150080, China

**Keywords:** monolithic integrated sensors, three-axis acceleration sensor, pressure sensor, magnetic field sensor, MEMS technology

## Abstract

In order to realize the measurement of three-axis acceleration, pressure, and magnetic field, monolithic integrated three-axis acceleration/pressure/magnetic field sensors are proposed in this paper. The proposed sensors were constructed with an acceleration sensor consisting of four L-shaped double beams, two masses, middle double-beams, and twelve piezoresistors, a pressure sensor made of a square silicon membrane, and four piezoresistors, as well as a magnetic field sensor composed of five Hall elements. COMSOL software and TCAD-Atlas software were used to simulate characteristics of integrated sensors, and analyze the working principles of the sensors in measuring acceleration, pressure, and magnetic field. The integrated sensors were fabricated by using micro-electro-mechanical systems (MEMS) technology and packaged by using inner lead bonding technology. When applying a working voltage of 5 V at room temperature, it is possible for the proposed sensors to achieve the acceleration sensitivities of 3.58 mV/g, 2.68 mV/g, and 9.45 mV/g along the *x*-axis, *y*-axis, and *z*-axis (through an amplifying circuit), and the sensitivities towards pressure and magnetic field are 0.28 mV/kPa and 22.44 mV/T, respectively. It is shown that the proposed sensors can measure three-axis acceleration, pressure, and magnetic field.

## 1. Introduction

Monolithic integrated sensors which share the same chip have attracted more and more attention due to their excellent properties for measuring different physical/chemical quantities. A variety of multifunctional integrated sensors are available [1], including integrated temperature/humidity/air velocity sensors [2], integrated pressure/temperature sensors [3,4], integrated accelerometers and magnetometers [5,6], etc., which can be used in a wide range of applications in industrial fields [7], wearable devices [8,9,10], robot environments [11,12], and so on. Dong et al. developed integrated acceleration and pressure multifunction sensors with a glass–silicon–glass sandwich structure based on piezoresistive effect, which can be applied to automobile, aerospace, environmental monitoring, and other application fields [13]. Wang et al. proposed composite sensors with pressure and two-axis acceleration that have potential in the mass production of tire pressure monitoring systems (TPMS) with a sensitivity of 0.136 mV/kPa [14]. With the recent expansion of the automotive electronic products market, and the increasing demand for sensors in the field of intelligent vehicles, sensors applied to the TPMS and intelligent driving field are developing rapidly. Among them, direct tire pressure sensors have received widespread attention due to their high accuracy and rapid response time [15,16]. Currently, light detection and ranging (LIDAR) [17,18] and visual image processing [19,20] are the most commonly used methods used by unmanned vehicles to recognize roads. At the entrance of most parking lots or garages there is a geomagnetic induction system, which has a buried geomagnetic induction coil that can generate magnetic field. When a vehicle passes or stops, the magnetic field in the environment changes. In this case, the magnetic field sensor on the vehicle can sense the change in the magnetic field and give feedback, which is used to assist road recognition in intelligent driving. Taking into account the above applications, the proposed integrated sensors can be applied to TPMS and can also assist road recognition in intelligent driving to provide a contribution to the future of the unmanned driving field.

In this paper, monolithic integrated sensors were presented so that three-axis acceleration, pressure, and magnetic field can be measured. The external acceleration and pressure are monitored through the piezoresistive effect, and the change in the external magnetic field is monitored through the Hall effect. The integrated sensors were fabricated by using micro-electro-mechanical systems (MEMS) technology on silicon-on-insulator (SOI) wafer and packaged by using inner lead bonding technology on a printed circuit board (PCB). In this study, the sensitivities, cross interference, and other characteristics of the sensors were evaluated. In terms of sensitivities, the proposed sensors exhibited a good performance, which is necessary for the use of the sensors in automotive electronics, tire pressure monitoring, etc. The study of monolithic integrated multifunctional sensors will be beneficial for the reduction of cross interference, improved performance, and wider applications in the future.

## 2. Basic Structure and Sensitive Characteristic Analysis

### 2.1. Basic Structure

**Figure 1:**
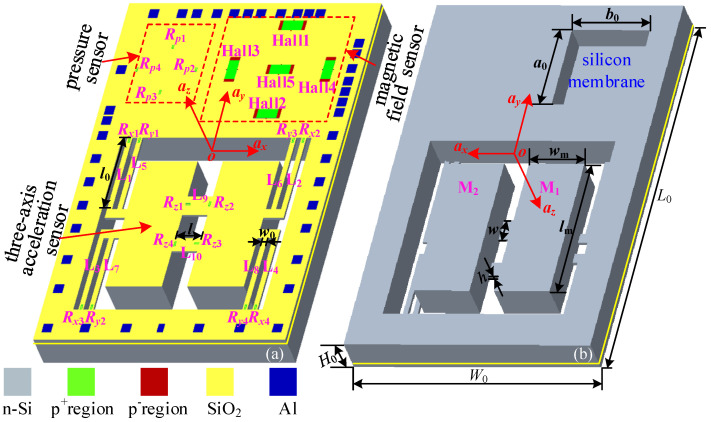
The basic structure of the integrated sensors: (**a**) top view; (**b**) back view.

The basic structure of the monolithic integrated sensors is proposed in this work, as shown in Figure 1, consisting of a three-axis acceleration sensor, a pressure sensor, and a magnetic field sensor. It can be seen that the three-axis acceleration sensor is made up of four L-shaped double beams (L_1_, L_2_, L_3_, L_4_, L_5_, L_6_, L_7_, L_8_), two masses (M_1_, M_2_), two middle beams (L_9_, L_10_), twelve piezoresistors, a support body, etc. As shown in Figure 1a, two Wheatstone bridges (W*_ax_*, W*_ay_*) along the *x* and *y* axes are prepared by eight piezoresistors (*R_x_*_1_, *R_x_*_2_, *R_x_*_3_, *R_x_*_4_, *R_y_*_1_, *R_y_*_2_, *R_y_*_3_, *R_y_*_4_) at the roots of the four L-shaped double beams. To make up the third Wheatstone bridge (W*_az_*) along the *z*-axis, the other four piezoresistors (*R_z_*_1_, *R_z_*_2_, *R_z_*_3_, *R_z_*_4_) are placed on the middle beams, respectively. The pressure sensor is composed of a square silicon membrane, four piezoresistors, etc. In addition, a Wheatstone bridge (W*_P_*) used to detect external pressure is also constituted by four piezoresistors (*R_p_*_1_, *R_p_*_2_, *R_p_*_3_, *R_p_*_4_) placed on the edge of the square silicon membrane. In which, Hall elements are used as a magnetic sensing unit, consisting of two Hall outputs (*V*_H1_, *V*_H2_) located in the middle of magnetic sensitive layer and two control current electrodes. The relevant dimensions are shown in Figure 1b and Table 1.

### 2.2. Sensitive Characteristic Analysis

#### 2.2.1. Simulation Analysis

**Figure 2:**
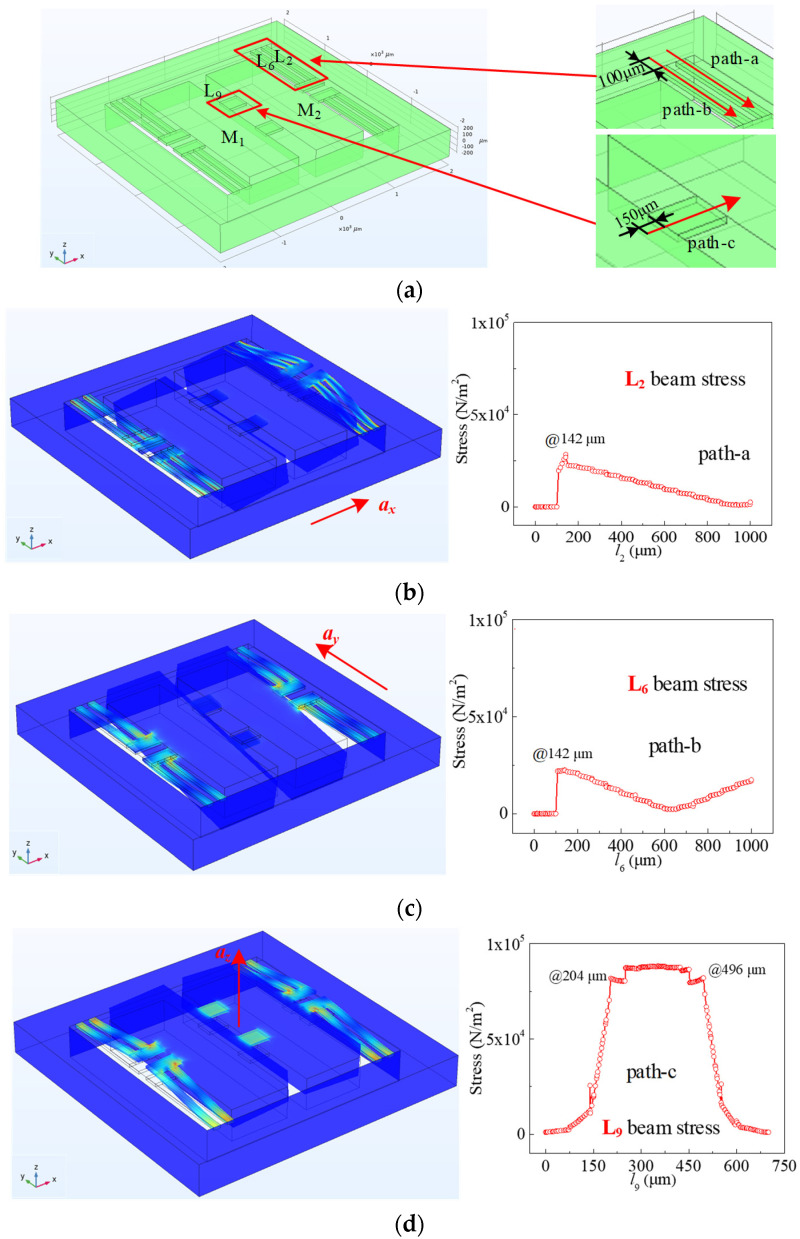
The deformation diagrams and stress distribution of three-axis acceleration sensor: (**a**) without acceleration; (**b**) the action of *a_x_*; (**c**) the action of *a_y_*; (**d**) the action of *a_z_*.

In this paper, COMSOL software (COMSOL Multiphysics® 5.0) was used to construct the simulation model and simulate the mechanical characteristics of the acceleration sensor and the pressure sensor. The simulation model for the magnetic field sensor was constructed by using TCAD-Atlas software (ATLAS commercial TCAD software 2014) and the magnetic characteristics were simulated. Figure 2a shows the simulation model for the acceleration sensor. The dimensions of the simulation model were designed according to the dimensions given in Section 2.1. During the process of simulation using the COMSOL software, an acceleration of 1 g was applied, respectively, along the *x*, *y*, and *z* axes through the gravitational field. Due to the symmetrical design of the three-axis acceleration sensor chip, as shown in Figure 2a, we utilized beams L_2_, L_6_, and L_9_ as examples to analyze the stress distribution under the action of acceleration. In order to design the piezoresistors for positions of high stress, the paths used to analyze stress were defined as shown in Figure 2a. The directions of the paths are along the arrow direction, where path-a along the beam is used to measure acceleration on the *x*-axis (*a_x_*), path-b along the beam is used to measure acceleration on the *y*-axis (*a_y_*), and path-c along the beam is used to measure acceleration on the *z*-axis (*a_z_*). Among them, the length of path-a, path-b, and path-c are 1000 μm, 1000 μm, and 700 μm. The starting points of path-a and path-b are located on the support body 100 μm away from the L-shaped double beam’s root. And the starting point of path-c is located on the mass (M_1_) 150 μm away from the middle beam’s root. The stress values on path-a, path-b, and path-c under the action of *a_x_*, *a_y_*, and *a_z_* were simulated, respectively, and the deformation diagrams were simulated as shown in Figure 2b–d. It is generally acknowledged that deviations in the two masses under the action of *a_x_*, *a_y_*, and *a_z_* would be caused according to Newton’s second law. As shown in Figure 2b, under the action of *a_x_*, the roots of L_1_ and L_3_ are subjected to tensile stress, and the roots of L_2_ and L_4_ are subjected to compressive stress. This leads to an increase in the resistance values of *R_x_*_1_ and *R_x_*_3_, while the resistance values of *R_x_*_2_ and *R_x_*_4_ decrease. Based on the above, *a_x_* can be measured by detecting the output voltages of W*_ax_* composed of *R_x_*_1_, *R_x_*_2_, *R_x_*_3_, and *R_x_*_4_. The detection principle of *a_y_* is the same as that of *a_x_*; that is, *a_y_* can be measured by detecting the output voltages of *W_ay_*. As shown in Figure 2d, under the action of *a_z_*, the roots of the middle beams are under great stress. *R_z_*_1_, *R_z_*_2_, *R_z_*_3_, and *R_z_*_4_ are designed on the roots of the middle beams, in which *R_z_*_1_ and *R_z_*_3_ are transverse resistors, *R_z_*_2_ and *R_z_*_4_ are longitudinal resistors. When the middle beams are subjected to tensile stress, the resistance values of *R_z_*_1_ and *R_z_*_3_ increase, and the resistance values of *R_z_*_2_ and *R_z_*_4_ decrease. Therefore, *a_z_* can be measured by detecting the output voltages of W*_az_* composed of *R_z_*_1_, *R_z_*_2_, *R_z_*_3_, and *R_z_*_4_.

**Figure 3:**
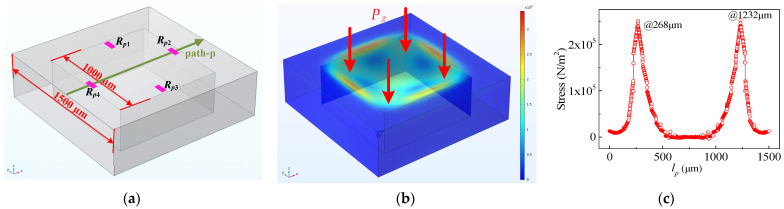
The deformation diagrams and stress distribution of pressure sensor: (**a**) in the absence of *P_z_*; (**b**) the deformation diagram under the action of *P_z_*; (**c**) stress distribution of path-p.

To realize the measurement of applied pressure (*P_z_*) perpendicular to the chip, the simulation model of the pressure sensor was constructed. It is shown in Figure 3a that the pressure sensor simulation model is symmetrical, and the relevant dimensions are given. The square silicon membrane on the chip deforms when external pressure is applied to it, resulting in changes in the piezoresistors. A pressure of 2 kPa was applied along the perpendicular direction of the chip, and the deformation diagram is shown in Figure 3b. The square silicon membrane deforms under the influence of *P_z_*, resulting in an enormous amount of stress at the center of the square silicon membrane’s edge. According to the piezoresistive effect, the piezoresistor should be placed at a position of high stress. In order to find the high stress position on the square silicon membrane, the path-p is set to simulate the stress distribution on it, as shown in Figure 3c. Because of the symmetrical design of the pressure sensor, the stress distribution is also symmetrical. Therefore, *R_p_*_1_, *R_p_*_2_, *R_p_*_3_, and *R_p_*_4_ are designed to be at the center of the square silicon membrane edge. Among them, *R_p_*_1_, *R_p_*_3_ are designed to be longitudinal resistances, and *R_p_*_2_, *R_p_*_4_ are designed to be horizontal resistances. When the square silicon membrane is affected by *P_z_*, *R_p_*_1_ and *R_p_*_3_ increase (or decrease) at the same time, the *R_p_*_2_ and *R_p_*_4_ also decrease (or increase) at the same time. Hence, *P_z_* can be measured by detecting the output voltages of W*_P_* composed of *R_p_*_1_, *R_p_*_2_, *R_p_*_3_, and *R_p_*_4_.

**Figure 4:**
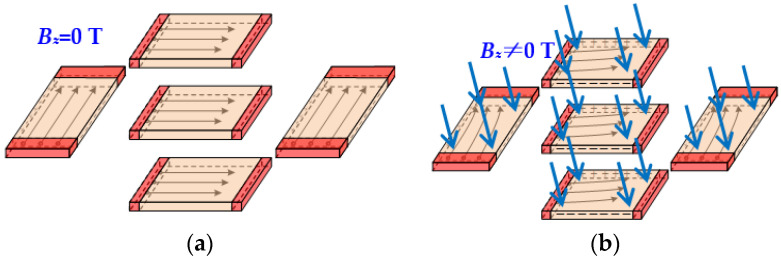
Working principle diagrams of magnetic field sensor: (**a**) *B_z_* = 0 T; (**b**) *B_z_* ≠ 0 T.

Based on the piezoresistive effect, p-type piezoresistors have better piezoresistive characteristics than n-type piezoresistors. Therefore, choosing n-type silicon to make p-type piezoresistors can improve their sensitivity towards the characteristics of acceleration and pressure. The sensitive elements of the magnetic field sensor belong to the Hall element, the magnetic sensitive layer fabricated on the p-type silicon is superior to the one on the n-type silicon. In order to ensure acceleration sensitivity and pressure sensitivity, n-type silicon was chosen in this paper. When a magnetic field (*B_z_*) is applied perpendicular to the surface of the magnetic field sensor, the carriers in the magnetic sensitive layer would be deflected by Lorentz force, leading to a potential difference between the two Hall output terminals, as shown in Figure 4.

**Figure 5:**
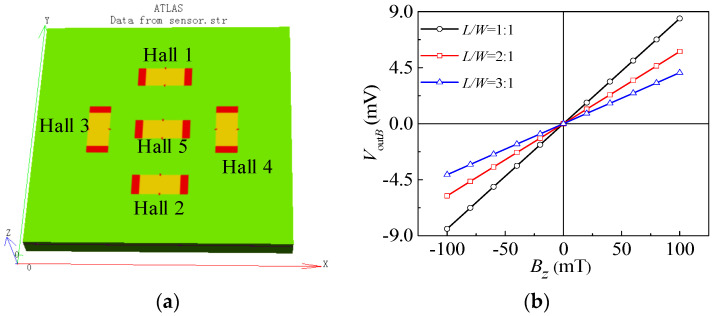
Simulation model and magnetic-sensitive characteristic curves of magnetic field sensor: (**a**) simulation model; (**b**) magnetic-sensitive characteristic curves.

Due to the fact that the length–width ratio of the Hall sensor’s sensitive region has a greater impact on its output voltage, we used TCAD simulation software to simulate the magnetic characteristics of the magnetic sensitive region with length–width ratios of 1:1, 2:1, and 3:1. The simulation model for the magnetic field sensor was constructed as shown in Figure 5a (length-width ratios of 2:1). The dimension of the magnetic-sensitive region is 320 μm × 160 μm, where the center of Hall 1, Hall 2, Hall 3, and Hall 4 are 760 μm away from the center of Hall 5. The impurity concentration for boron-ion implantation was set to 1.0 × 10^14^ cm^−3^. With a 5 V working voltage, the range of *B_z_* was between −100 mT and 100 mT, and the step was 20 mT. Taking Hall 3 as an example, Figure 5b shows the relationship curves between the output voltages and *B_z_*. It can be seen that the output voltage of the magnetic field sensor increases as the *B_z_* increases. Therefore, the magnetic field sensor can detect the magnetic field perpendicular to the surface of the chip. And the slope of characteristic curve is higher under 1:1 length-width ratio. Since smaller length-width ratio may result in a stronger short-circuiting effect, a length–width of 2:1 was chosen for the fabrication process in this paper.

#### 2.2.2. Sensitivity Analysis

**Figure 6:**
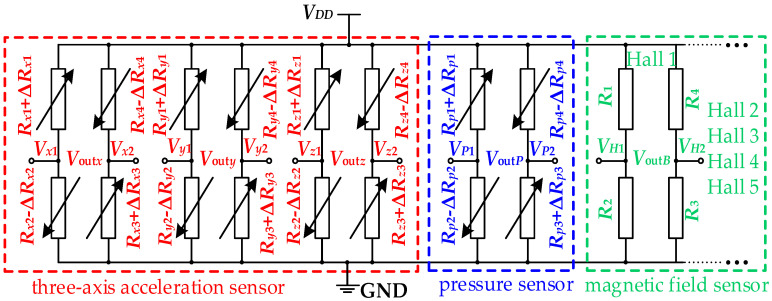
The equivalent circuit of the three-axis acceleration/pressure/magnetic field sensors.

In order to study the sensitivity characteristics of the proposed sensors, the equivalent circuit is given in Figure 6. The equivalent circuit of acceleration sensor consists of three Wheatstone bridges (W*_ax_*, W*_ay_*, and W*_az_*) composed of twelve piezoresistors. In ideal circumstances, the resistances of the piezoresistors are equal; that is, *R_x_*_1_ = *R_x_*_2_ = *R_x_*_3_ = *R_x_*_4_ = *R_x_*, *R_y_*_1_ = *R_y_*_2_ = *R_y_*_3_ = *R_y_*_4_ = *R_y_*, *R_z_*_1_ = *R_z_*_2_ = *R_z_*_3_ = *R_z_*_4_ = *R_z_.* Based on the piezoresistive effect, when piezoresistors are subjected to stress, the resistance value of the piezoresistors changes. When other factors are ignored, the relative variations in the piezoresistors are equal, respectively, under the action of *a_x_*, *a_y_*, and *a_z_*; that is, |∆*R_x_*_1_| = |−∆*R_x_*_2_| = |∆*R_x_*_3_| = |−∆*R_x_*_4_| = ∆*R_x_*, |∆*R_y_*_1_| = |−∆*R_y_*_2_| = |∆*R_y_*_3_| = |−∆*R_y_*_4_| = ∆*R_y_*, |∆*R_z_*_1_| = |−∆*R_z_*_2_| = |∆*R_z_*_3_| = |−∆*R_z_*_4_| = ∆*R_z_*. The output voltages of W*_ax_*, W*_ay_*, and W*_az_* change with the acceleration along the sensitive axes, responding to *a_x_*, *a_y_*, and *a_z_*, respectively. From the above analysis and the working principle of a Wheatstone bridge, the *V*_out*x*_, *V*_out*y*_, and *V*_out*z*_ can be expressed as

(1) $$\left\{ \begin{array}{l} V_{\mathrm{out}x}=V_{x2}-V_{x1}=\left(\frac{R_{x3}+\Delta R_{x3}}{R_{x3}+\Delta R_{x3}+R_{x4}-\Delta R_{x4}}-\frac{R_{x2}-\Delta R_{x2}}{R_{x1}+\Delta R_{x1}+R_{x2}-\Delta R_{x2}}\right)\cdot V_{DD}=\frac{\Delta R_{x}}{R_{x}}\cdot V_{DD}\\ V_{\mathrm{out}y}=V_{y2}-V_{y1}=\left(\frac{R_{y3}+\Delta R_{y3}}{R_{y3}+\Delta R_{y3}+R_{y4}-\Delta R_{y4}}-\frac{R_{y2}-\Delta R_{y2}}{R_{y1}+\Delta R_{y1}+R_{y2}-\Delta R_{y2}}\right)\cdot V_{DD}=\frac{\Delta R_{y}}{R_{y}}\cdot V_{DD}\\ V_{\mathrm{out}z}=V_{z2}-V_{z1}=\left(\frac{R_{z3}+\Delta R_{z3}}{R_{z3}+\Delta R_{z3}+R_{z4}-\Delta R_{z4}}-\frac{R_{z2}-\Delta R_{z2}}{R_{z1}+\Delta R_{z1}+R_{z2}-\Delta R_{z2}}\right)\cdot V_{DD}=\frac{\Delta R_{z}}{R_{z}}\cdot V_{DD} \end{array}\right.$$

where *V_DD_* is the supply voltage.

As shown in Figure 6, the equivalent circuit of the pressure sensor is a Wheatstone bridge (W*_P_*) composed of four piezoresistors. In ideal circumstances, the resistances of piezoresistors are equal, that is, *R_p_*_1_ = *R_p_*_2_ = *R_p_*_3_ = *R_p_*_4_ = *R_p_.* Under the action of *P_z_*, the silicon membrane deforms and the resistances of the piezoresistors change. When other factors are ignored, the relative variations in the piezoresistors are equal, that is, |∆*R_p_*_1_| = |−∆*R_p_*_2_| = |∆*R_p_*_3_| = |−∆*R_p_*_4_| = ∆*R_p_*. According to the working principle of a Wheatstone bridge, the variation in the output voltage (*V*_out*P*_) with *P_z_* can be realized through the equivalent circuit of the pressure sensor. The *V*_out*P*_ of W*_P_* can be expressed as

(2) $$V_{\mathrm{out}P}=V_{P2}-V_{P1}=\left(\frac{R_{p3}+\Delta R_{p3}}{R_{p3}+\Delta R_{p3}+R_{p4}-\Delta R_{p4}}-\frac{R_{p2}-\Delta R_{p2}}{R_{p1}+\Delta R_{p1}+R_{p2}-\Delta R_{p2}}\right)\cdot V_{DD}=\frac{\Delta R_{p}}{R_{p}}\cdot V_{DD}$$

The magnetic field sensor in this paper was composed of Hall elements with a magnetic-sensitive layer made of n-type silicon; the control current electrode and the Hall output can be equivalent to the variable resistor. Since the Hall element is a four-ended element, it can be equivalent to four variable resistors, as shown in Figure 6. Under the action of *B_z_*, the holes in the magnetic-sensitive layer are deflected by the Lorentz force. Thereby the output voltage of the Hall element (*V*_out*B*_) changes with *B_z_*, achieving the measurement of *B_z_*. Based on Hall effect, the *V*_out*B*_ as follows [21]:

(3) $$V_{\mathrm{out}B}=V_{H1}-V_{H2}=\mu_{p}\cdot \frac{W}{L}\cdot V_{DD}\cdot B_{z}\cdot f_{H}\left(\frac{L}{W},\theta \right)$$

where $f_{H}\left(\frac{L}{W},\theta \right)$ is the geometrical factor. *L* and *W* are the length and width of the magnetic-sensitive region, respectively. *μ_p_* is the hole mobility.

As can be seen from Equations (1)–(3), when there is no external acceleration, pressure, or magnetic field, the output voltages of the proposed sensors are zero under ideal conditions. Furthermore, this indicates that the output voltages of the proposed sensors will change under the action of *a_x_*, *a_y_*, *a_z_*, *P_z_*, and *B_z_*, thus it has sensitive characteristics. According to the definition of sensor sensitivity, we defined the sensitivities of the proposed sensors; that is, *S_xx_*, *S_yy_*, *S_zz_*, *S_PP_*, and *S_BB_* are the sensitivities of *a_x_*, *a_y_*, *a_z_*, *P*, and *B*, respectively. However, in the process of chip operation, there may be cross interference, which includes cross interference among three axes during acceleration measurement and cross interference during the measurement of acceleration, pressure, and magnetic field. In this paper, the cross sensitivities are defined so that *S_yx_*, *S_zx_*, *S_Px_*, and *S_Bx_* are the cross sensitivities at *a_x_*; *S_xy_*, *S_zy_*, *S_Py_*, and *S_By_* are the sensitivities at *a_y_*, *S_xz_*, *S_yz_*, *S_Pz_*, and *S_Bz_* are those at *a_z_*. *S_xP_*, *S_yP_*, *S_Pz_*, and *S_BP_* are the cross sensitivities at *P_z_*. Correspondingly, *S_xB_*, *S_yB_*, *S_zB_*, and *S_PB_* are their cross sensitivities under the action of *B_z_*. The output voltages of the integrated sensors can be expressed as

(4) $$\left[\begin{array}{l} V_{\mathrm{out}x}\\ V_{\mathrm{out}y}\\ V_{\mathrm{out}z}\\ V_{\mathrm{out}P}\\ V_{\mathrm{out}B} \end{array}\right]=\left[\begin{array}{ccccc} S_{xx} & S_{xy} & S_{xz} & S_{xP} & S_{xB}\\ S_{yx} & S_{yy} & S_{yz} & S_{yP} & S_{yB}\\ S_{zx} & S_{zy} & S_{zz} & S_{zP} & S_{zB}\\ S_{Px} & S_{Py} & S_{Pz} & S_{PP} & S_{PB}\\ S_{Bx} & S_{By} & S_{Bz} & S_{BP} & S_{BB} \end{array}\right]\left[\begin{array}{l} a_{x}\\ a_{y}\\ a_{z}\\ P_{z}\\ B_{z} \end{array}\right]$$

As can be seen from Equation (4), the cross sensitivities will affect the characteristics of the proposed sensors. However, the cross sensitivities can be reduced by optimizing the structural dimensions, process conditions, etc. Under ideal conditions, if the cross interference can be ignored, it is as follows.

(5) $$\left[\begin{array}{l} V_{\mathrm{out}x}\\ V_{\mathrm{out}y}\\ V_{\mathrm{out}z}\\ V_{\mathrm{out}P}\\ V_{\mathrm{out}B} \end{array}\right]=\left[\begin{array}{ccccc} S_{xx} & 0 & 0 & 0 & 0\\ 0 & S_{yy} & 0 & 0 & 0\\ 0 & 0 & S_{zz} & 0 & 0\\ 0 & 0 & 0 & S_{PP} & 0\\ 0 & 0 & 0 & 0 & S_{BB} \end{array}\right]\left[\begin{array}{l} a_{x}\\ a_{y}\\ a_{z}\\ P_{z}\\ B_{z} \end{array}\right]$$

## 3. Fabrication of the Technology for the Integrated Sensors

**Figure 7:**
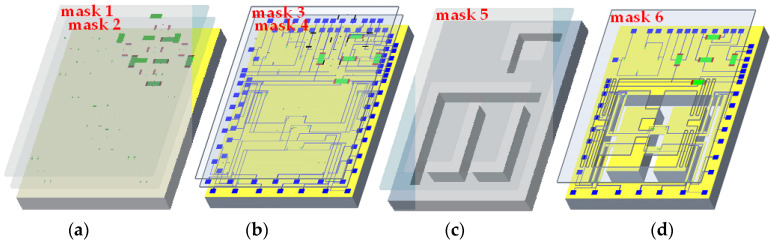
Main fabrication processes of the chip: (**a**) cleaning SOI wafer, growing SiO_2_, and forming p+ region and p− region; (**b**) metallization to form pad and aluminum leads; (**c**) etching the back side to form silicon cup and masses; (**d**) releasing beam structure on surface.

According to the analysis of the structure of the proposed sensors and the simulation results, the proposed sensors were fabricated by using MEMS technology. An SOI wafer with n-type <100> orientation in the device layer was selected. The whole process of creating the proposed sensors went through photolithography six times. The main fabrication processes are shown in Figure 7.

**Figure 8:**
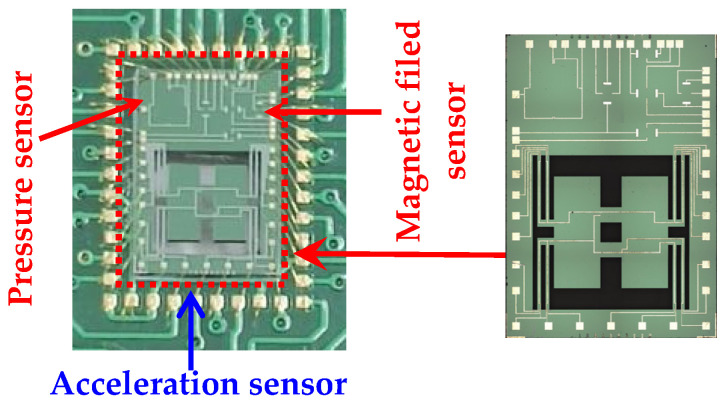
Photograph of proposed sensors.

First of all, the SOI wafer was cleaned with RCA standard cleaning and an SiO_2_ layer was grown by thermal oxidation on its surface. In the first photolithography, 1 × 10^14^ cm^−2^ boron ions were implanted using mask 1 at an energy of 60 keV. In the second photolithography, 1 × 10^16^ cm^−2^ boron ions were implanted using mask 2 at an energy of 60 keV. The piezoresistors and Hall elements were manufactured by the above processes shown in Figure 7a. The width of the piezoresistors was set to 10 μm according to the process conditions. Then, an SiO_2_ layer was grown on the surface of the SOI wafer, and mask 3 was used for the third photolithography to form the lead holes. After that, an aluminum layer was formed on the surface of the SOI wafer by vapor deposition, and mask 4 was used for the fourth photolithography to form aluminum leads and pads as shown in Figure 7b. After that, mask 5 was used for the fifth photolithography, and inductively coupled plasma (ICP) technology was used to etch the back of the SOI wafer to form a silicon cup and masses, as shown in Figure 7c. Mask 6 was used for the sixth photolithography as well as ICP technology to release the beams as shown in Figure 7d. The wafer with integrated sensors was obtained through the above process flow. The finished wafer was divided by the scribing machine (D2610, Heyan Technology, Hebei, China), and the chips were selected by the microscope (STM7-SF, Olympus Corporation, Tokyo, Japan) and the electrical characteristic test, etc. After that, the selected chips were packaged on the PCB by utilizing inner lead bonding technology to test for the sensitive characteristics. A photograph of the packaged sensor is shown in Figure 8.

## 4. Results and Discussion

### 4.1. Test Systems of Integrated Sensors

**Figure 9:**
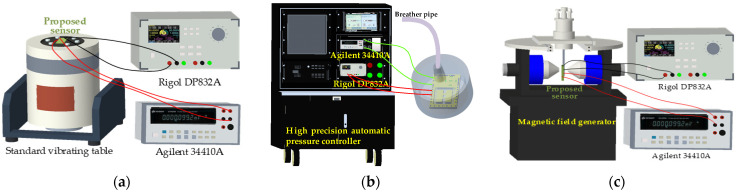
Test systems of integrated sensors: (**a**) test system for acceleration sensor; (**b**) test system for pressure sensor; (**c**) test system for magnetic field sensor.

In order to examine the sensitive characteristics of three-axis acceleration, pressure, magnetic field sensor as well as cross-interference, test systems were provided, as shown in Figure 9. The acceleration sensor was calibrated by the standard vibrating table (ESS-050, Suzhou Dongling Vibration Test Instrument Co., Ltd., Suzhou, China) test system shown in Figure 9a. The pressure sensor was measured by the high precision automatic pressure controller (CPC6000, Beijing Lesentech Technology Co., Ltd, Beijing, China) test system shown in Figure 9b. The magnetic field sensor was tested using the magnetic field generator (CH100, Beijing Cuihai Jiacheng Magnetic Technology Co., Ltd., Beijing, China) test system shown in Figure 9c. The whole test process was carried out at room temperature and a relative humidity of 20% RH. And the integrated sensors were powered by programmable linear direct-current power (DP832A, Rigol Technologies Co., Ltd., Beijing, China). The output voltages of the integrated sensors were collected by digital multimeter (34410A, Agilent Technologies Inc., New York, NY, USA).

### 4.2. Sensitive Characteristics of the Three-Axis Acceleration Sensor

**Figure 10:**
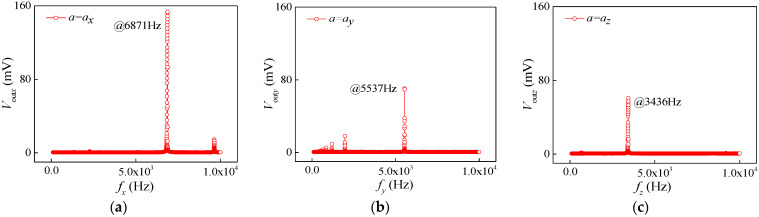
Resonance characteristics of acceleration sensor: (**a**) *a* = *a_x_*; (**b**) *a* = *a_y_*; (**c**) *a* = *a_z_*.

Both the proposed packaged chip and the standard accelerometer were rigidly mounted on the standard vibrating table. To begin with, the frequency response characteristics of the three-axis acceleration sensor were analyzed. Under an operating voltage of 5 V and a constant acceleration of 0.4 g, the output voltages of the sensor were tested when the vibration frequency changed within the range of 0–10,000 Hz. As shown in Figure 10a–c, the relationship curves of the output voltage vs. the excitation frequency were plotted, representing the resonance characteristics along the *x*-axis, *y*-axis, and *z*-axis, respectively. When applying *a_x_*, the masses produced displacement, causing the L-shaped double beams to be squeezed or stretched. When increasing the frequency of *a_x_* up to a certain value, the L-shaped double beams reached the maximum deformation by resonating, resulting in the maximum stress at the root of the L-shaped double beams. Meanwhile, the W*_ax_* output a maximum voltage. As shown in Figure 10a, the output voltage changes with the increasing excitation frequency. In particular, the output voltages would be rapidly increase with the addition of a frequency along the *x*-axis close to 6871 Hz. When continually increasing the excitation frequency to more than 6871 Hz, the output voltages begin to rapidly decrease. From the above analysis, it can be found that the resonant frequency along the *x*-axis is 6871 Hz. Due to the fact that both the *y*-axis and *z*-axis measure acceleration in similar principle to the *x*-axis, the resonant frequencies along the *y*-axis and *z*-axis had the values of 5537 Hz and 3436 Hz, as shown in Figure 10b,c.

**Figure 11:**
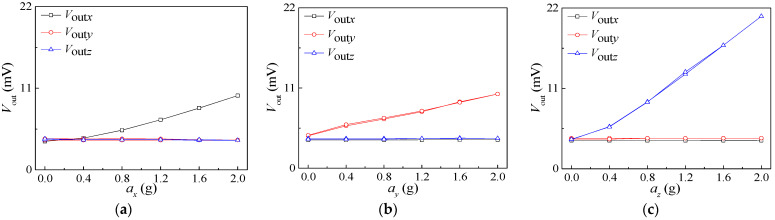
The characteristic curves of acceleration sensor: (**a**) *a* = *a_x_*; (**b**) *a* = *a_y_*; (**c**) *a* = *a_z_*.

Due to the output voltages of the three-axis acceleration sensor being small at the non-resonant frequency, the output voltages were amplified by differential amplifier, and the relationships between *V*_out*x*_, *V*_out*y*_, *V*_out*z*_ and acceleration were studied. During the test process an external acceleration step of 0.4 g was varied within the range of 0 g to 2 g with a 5 V supply voltage. The above test was repeated three times for forward and reverse strokes, and average values were taken to draw characteristic curves, as shown in Figure 11a–c, respectively.

It can be seen from Figure 11 that the output voltages of the Wheatstone bridges along the sensitive axis increase with the applied acceleration, while the output voltages of the Wheatstone bridges along the non-sensitive axis remain practically unchanged with the increase in applied acceleration. A comparison of Figure 11c with Figure 11a,b reveals that the slope of the sensitive axis in Figure 11c is higher. On the basis of the simulation results in Figure 2b–d, the stress on the sensitive beam in the *z*-axis direction is greater under *a_z_*, so the acceleration sensitivity in the *z*-axis direction is greater than that in the *x*-axis and *y*-axis directions. In view of Equation (4), the *S_xx_*, *S_yy_*, and *S_zz_* can be calculated, i.e., 3.577 mV/g, 2.678 mV/g, and 9.452 mV/g, respectively. This indicates that the proposed sensor can measure *a_x_*, *a_y_*, and *a_z_* with low cross interference.

### 4.3. Sensitive Characteristics of the Pressure Sensor

**Figure 12:**
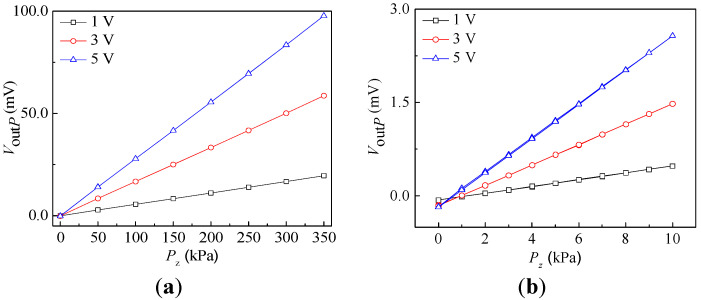
Input and output characteristic curves of pressure sensor: (**a**) 0–350 kPa; (**b**) 0–10 kPa.

During the testing process, the chip was placed inside a sealed tube seat. The current tire pressure used for small cars is between 210 kPa and 240 kPa, and that used for large cars is between 260 kPa and 310 kPa. In this application scenario, the pressure sensor’s characteristics were tested under pressure perpendicular to the chip surface at levels ranging from 0 kPa to 350 kPa in steps of 50 kPa. The static characteristics of the pressure sensor can be measured by exerting supply voltages of 1 V, 3 V, and 5 V, respectively. The test was repeated three times, and average values were taken to draw the testing curves of the *V*_out*P*_ vs. *P_z_* shown in Figure 12a. In addition, in order to observe the characteristics of the pressure sensor in the pressure range of 0–10 kPa, we also carried out the characteristics test under the same conditions at 0–10 kPa with a step of 1 kPa as shown in Figure 12b.

It can be found that, under the same supply voltages, the *V*_out*P*_ increases with *P_z_* at pressure ranges of 0–350 kPa and 0–10 kPa as seen in Figure 12a,b. The slope of curve is bigger than the others at a supply voltage of 5 V. The *S_PP_* of the pressure range 0–350 kPa at supply voltages of 1 V, 3 V, and 5 V were calculated, i.e., 0.056 mV/kPa, 0.167 mV/kPa, and 0.278 mV/kPa. The *S_PP_* of the pressure range 0–10 kPa at the same supply voltages were 0.054 mV/kPa, 0.164 mV/kPa, and 0.275 mV/kPa, respectively. The pressure sensitivities in the pressure range 0-350 kPa and 0-10 kPa are not equal, mainly due to the nonlinearity of the pressure sensor. According to Equation (2), the *V*_out*P*_ should be proportional to the supply voltages. However, the pressure sensitivities under the three supply voltages are not proportional. This may be caused by the deviation in the test system, power consumption during operation, etc. Moreover, the nonlinearities of the pressure sensor at the same supply voltages were obtained using the least square method, with a full range of outputs (0–350 kPa) of 0.41%, 0.43%, and 0.43%, respectively. In terms of the above calculated sensitivities and nonlinearities, it is clear that the proposed pressure sensor not only achieves the pressure measurement but also has better linearity.

### 4.4. Sensitive Characteristics of Magnetic Field Sensor

**Figure 13:**
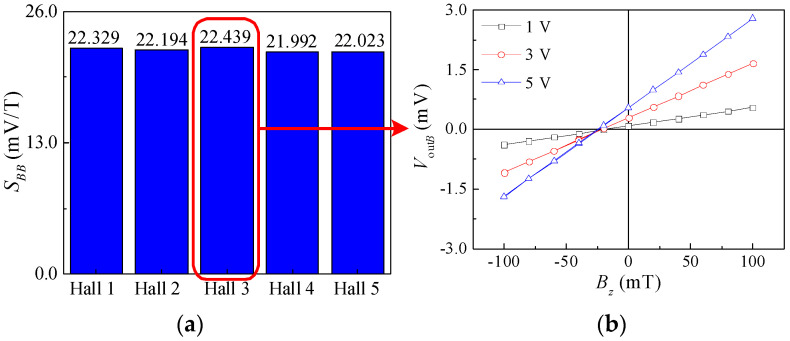
Magnetic sensitive characteristic diagrams of magnetic field sensor: (**a**) sensitivities of five Hall elements; (**b**) input and output characteristic curves of Hall 3.

The proposed sensor was placed in the magnetic field generator, and the sensitive direction of the magnetic field sensor was consistent with the direction of the magnetic field. The magnetic field changed from −100 mT to 100 mT and then from 100 mT to −100 mT with a step of 20 mT as a cycle with working voltage of 5 V. The experiment was carried out three times in succession. Five Hall elements in series were tested. The sensitivities of the five Hall elements were calculated as 22.329 mV/T, 22.194 mV/T, 22.439 mV/T, 21.992 mV/T, and 22.023 mV/T, as shown in Figure 13a. The *V*_out*B*_ vs. *B_z_* at different supply voltages is given for Hall 3 as an example, as shown in Figure 13b. It can be seen that *V*_out*B*_ is approximately proportional to *B_z_* at a constant supply voltage. Under constant *B_z_*, the *V*_out*B*_ increases with the supply voltage. When the supply voltages are 1 V, 3 V, and 5 V, the magnetic sensitivities are 4.644 mV/T, 13.643 mV/T, and 22.439 mV/T, respectively. Compared with the simulation results, the experimental magnetic sensitivity is low, mainly because of the poor ohmic contact and uneven doping concentration in the process of fabrication and so on. The proposed magnetic field sensor also exhibits good linearity, as shown in Figure 13b.

### 4.5. The Mutual Interference of Integrated Sensors

**Figure 14:**
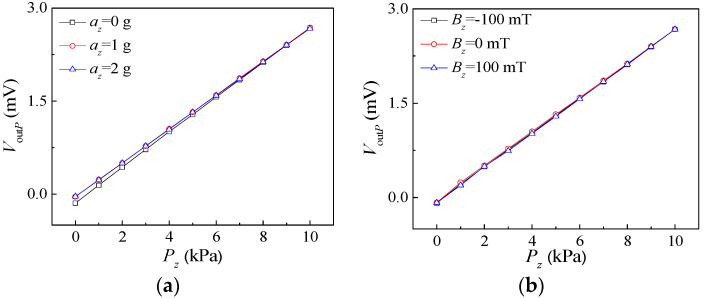
The relationship curves of pressure sensor under the action of *a_z_* and *B_z_*: (**a**) the action of *a_z_*; (**b**) the action of *B_z_*.

To investigate the mutual interference of the integrated sensors, the characteristics of the pressure sensor under the influence of *a_z_* and *B_z_* were analyzed. And the output characteristics of acceleration sensor and magnetic field sensor acting on *P_z_* were analyzed. A sealed tube seat with a packaged chip was placed on the standard vibrating table through a rigid connection and accelerations of 1 g and 2 g were applied, respectively. Figure 14a shows the relationship curves of *V*_out*P*_ vs. *P_z_* at a supply voltage of 5 V under pressures from 0 kPa to 10 kPa with a step of 1 kPa. Under the conditions of *a_z_*, the slopes of the input and output characteristic curves for the pressure sensor are smaller than under the condition of no external acceleration. In this situation, it is possible that the square silicon membrane of the pressure sensor vibrates under the influence of *a_z_*, which affects the output voltages. It can be concluded from the Figure 14a and above analysis that acceleration has a slight impact on the pressure sensor’s characteristics if the acceleration is applied appropriately. The sealed tube seat with the proposed sensor was placed in the magnetic field generator, and the sensitive direction of the magnetic field sensor was consistent with the direction of the magnetic field. Relationship curves of *V*_out*P*_ vs. *P_z_* when exerting *B_z_* with −100 mT and 100 mT are shown in Figure 14b. As can be seen from the figure, there is only a slight deviation between the three input and output characteristic curves. In the above performance tests, the magnetic field has little effect on the characteristics of the pressure sensor. In conclusion, it is clear that both acceleration and magnetic field have a very small impact on the detection of pressure when other influencing factors are ignored.

**Figure 15:**
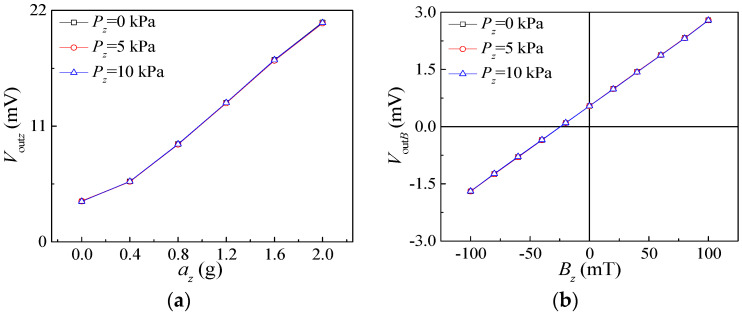
Input and output characteristic curves of acceleration sensor and magnetic field sensor under the action of *P_z_*: (**a**) input and output characteristic curves of acceleration sensor; (**b**) input and output characteristic curves of magnetic field sensor.

The packaged chip with a sealed tube seat was fixed respectively to the standard vibrating table and the magnetic field generator. The effects of *P_z_* on the characteristics of the acceleration sensor and the magnetic field sensor were studied as shown in Figure 15. The cross interference of sensors was analyzed by applying *P_z_* at 5 kPa and 10 kPa under the supply voltage of 5 V. The relationship curves of *V*_out*z*_ vs. *a_z_* are shown in Figure 15a. The range of *a_z_* was from 0 g to 2 g with a step of 0.4 g. The figure illustrates that the three input and output characteristic curves are essentially in agreement. This shows that the *P_z_* has a slight effect on the acceleration sensor. The relationship curves of *V*_out*B*_ vs. *B_z_* under applied *P_z_* are shown in Figure 15b. The range of applied magnetic field was from −100 mT to 100 mT with a step of 20 mT. The experimental curves indicate that the relationship curves of *V*_out*B*_ vs. *B_z_* remain basically unchanged when *P_z_* is applied. This indicates that the applied pressure has few impacts on the detection of the magnetic field.

To sum up, the magnetic field has little influence on the sensitivity characteristic of the pressure sensor. Nevertheless, applied acceleration has a small effect on the sensitivity characteristic of the pressure sensor. The applied pressure has less effect on the sensitivity characteristics of the acceleration sensor and the magnetic field sensor. Besides, in order to study the non-sensitive characteristics of the proposed sensors under the action of *a_x_*, *a_y_*, *a_z_*, *P_z_*, and *B_z_*, we tested the output voltages of the non-sensitive quantity with *a_x_*, *a_y_*, *a_z_*, *P_z_*, and *B_z_* applied respectively under the supply voltage of 5 V. The sensitivities and cross sensitivities of the integrated sensors were calculated according to the sensitivity definition, as shown in Table 2.

The first subscript in *S_xn_*, *S_yn_*, *S_zn_*, *S_Pn_*, and *S_Bn_* of Table 2 represents the sensitive physical quantities corresponding to the sensor, and the second subscript represents the external physical quantities applied to the sensor, i.e., *a_x_*, *a_y_*, *a_z_*, *P_z_*, and *B_z_*. When the physical quantities of the first and second subscripts are the same, it represents the sensitivities of the proposed sensors in the corresponding sensitivity quantity. When the physical quantities of the first and second subscripts are different, it represents the cross sensitivities of the non-sensitive physical quantity under the action of *a_x_*, *a_y_*, *a_z_*, *P_z_*, and *B_z_*. The comparison of the data shown in Table 2 with Equation (4) shows that the proposed sensors have certain cross influences between different physical quantities, but the cross sensitivities are small. The main reasons include the deviations in structure design and fabrication process of proposed sensors, the time drifting of output voltages, the deviations during the test process, and so on. In the future, we will further analyze the main factors affecting cross interference, and reduce the impact of cross interference on sensitive characteristics of proposed sensors as much as possible.

## 5. Conclusions

In summary, monolithic integrated acceleration/pressure/magnetic field sensors based on MEMS technology were fabricated on an SOI wafer with n-type silicon device layer in this paper. As the experimental results demonstrate, the proposed sensor can realize the detection of three-axis acceleration, pressure, and magnetic field. Moreover, it is evident from the cross characteristic experimental results that there is less cross interference when measuring acceleration in three axes. Similarly, when measuring acceleration, pressure, and magnetic field, there is also less cross interference between them. The study of integrated sensors will be helpful for their future applications in the fields of automotive electronics, intelligent driving, etc.

## Figures and Tables

**Table 1:** Dimension parameters of the integrated sensors.

Elements	Parameters	Values
L-shaped double beams (L_1_, L_2_, L_3_, L_4_, L_5_, L_6_, L_7_, L_8_)	*l*_0_, *w*_0_	1250 μm, 100 μm
Middle beams (L_9_, L_10_)	*l*, *w*	400 μm, 250 μm
Mass (M_1_, M_2_)	*l*_m_, *w*_m_	2160 μm, 900 μm
Square silicon membrane	*a*_0_, *b*_0_	1000 μm, 1000 μm
Thicknesses of beam and square silicon membrane	*h*	40 μm

**Table 2:** Sensitivities and cross sensitivities of integrated packaged sensors.

	*a_x_* (g)	*a_y_* (g)	*a_z_* (g)	*P_z_* (kPa)	*B_z_* (T)
*S_xn_*	3.577	0.014	0.004	−0.002	−0.194
*S_yn_*	−0.040	2.678	0.038	−0.005	−0.072
*S_zn_*	−0.055	0.049	9.452	0.002	−0.003
*S_Pn_*	0.008	0.011	0.009	0.275	−0.022
*S_Bn_*	0.001	−0.002	0.002	3 × 10^−4^	22.439

The second subscript-*n* in *S_xn_*, *S_yn_*, *S_zn_*, *S_Pn_*, and *S_Bn_* can be represented respectively as *x*, *y*, *z*, *P*, and *B* as shown in Equation (4). When *n* is represented as *x*, *y*, and *z*, the units of sensitivities and cross sensitivities are mV/g; when *n* is represented as *P*, the units of sensitivities and cross sensitivities are mV/kPa; when *n* is represented as *B*, the units of sensitivities and cross sensitivities are mV/T.

## Data Availability

Data are contained within the article.
